# Frontal plane biomechanics during single-leg squat and hip strength in patients with isolated patellofemoral osteoarthritis compared to matched controls: A cross-sectional study

**DOI:** 10.1371/journal.pone.0267446

**Published:** 2022-04-27

**Authors:** Cristiano Carvalho, Fábio Viadanna Serrão, Giulia Keppe Pisani, Adalberto Felipe Martinez, Paula Regina Mendes da Silva Serrão

**Affiliations:** 1 Department of Physical Therapy, Federal University of São Carlos, São Carlos, SP, Brazil; 2 Physical Therapy Post-Graduate Program, Federal University of São Carlos, São Carlos, SP, Brazil; The Wingate College of Physical Education and Sports Sciences at the Wingate Institute, IL, ISRAEL

## Abstract

The patellofemoral compartment of the knee is the most frequently affected by osteoarthritis. However, there is a lack of biomechanics studies on patellofemoral osteoarthritis (PFOA). This study’s purpose was to compare the frontal plane biomechanics of the trunk and lower limb during the single-leg squat and isometric hip abductor torque in individuals with isolated PFOA and controls. Frontal plane kinematics during the single-leg squat were evaluated using a three-dimensional (3-D) motion analysis system. Isometric hip abductor torque was determined using a handheld dynamometer. Twenty individuals participated in the study (10 with PFOA and 10 controls). No significant differences between groups were found regarding age (mean ± SD, PFOA group = 51.8 ± 6.9 versus control group = 47.8 ± 5.5; mean difference = 4, 95% confidence interval [CI] = -1.9 to 9.9, p = 0.20) or body mass index (PFOA group = 27.6 ± 2.2 versus control group = 25.5 ± 2.5; mean difference = 2.1, 95% confidence interval [CI] = -0.1 to 4.3, p = 0.06). Compared to control, the PFOA group presented greater hip adduction in the descending and ascending phases of the single-leg squat at 45° (mean difference [95% CI] = 6.44° [0.39–12.48°], p = 0.04; mean difference [95% CI] = 5.33° [0.24–10.42°], p = 0.045, respectively) and 60° (mean difference [95% CI] = 8.44° [2.15–14.73°], p = 0.01; mean difference [95% CI] = 7.58° [2.1–13.06°], p = 0.009, respectively) of knee flexion. No significant differences between groups were found for the frontal plane kinematics of the trunk, pelvis or knee (p > 0.05). The PFOA group exhibited lower isometric hip abductor torque (mean difference [95% CI] = -0.34 Nm/kg [-0.67 to -0.01 Nm/kg], p = 0.04). The individuals with PFOA presented greater hip adduction than the control group, which could increase lateral patellofemoral joint stress at 45° and 60° of knee flexion in the descending and ascending phases of the single-leg squat. These individuals also exhibited hip abductor weakness in comparison to healthy controls.

## Introduction

The patellofemoral compartment of the knee is the most frequently affected by osteoarthritis (OA) [1,2], which is associated with considerable pain and functional limitations during activities of daily living [3–6]. However, studies on OA of the knee focus mainly on individuals with OA in the tibiofemoral compartment or both compartments, whereas isolated patellofemoral osteoarthritis (PFOA) is studied little.

Consequently, the literature on biomechanics associated with tibiofemoral OA is vast, especially OA in the medial tibiofemoral compartment. This preference likely reflects the greater incidence of the condition, as activities of daily living often involve weight-bearing loads, as occurs during gait. Throughout the gait cycle, an external adduction moment tends to rotate the tibia medially in relation to the femur [7–10]. Such mechanics lead to the compression of the tibiofemoral compartments, especially the medial compartment [11,12]. Thus, a high knee adduction moment reflects an increase in compressive forces on the medial face of the knee [13,14], which may be one of the predisposing factors for the emergence of OA in these compartments. However, data on biomechanical characteristics in individuals with isolated PFOA are scarce.

As patellofemoral pain (PFP) is speculated to be a precursor of PFOA [6,15,16], altered biomechanics may be similar between the two conditions. Studies have reported an increase in hip adduction, knee abduction, contralateral pelvic drop and ipsilateral trunk lean in individuals with PFP during functional activities involving body weight bearing compared to healthy individuals [17–19]. The increase in these movements on the frontal plane can result in an increase in patellofemoral stress [20–22].

As occurs in PFP, some studies have found an increase in hip adduction during gait and knee abduction during the task of sit to stand in individuals with PFOA compared to healthy controls [23,24]. On the other hand, a recent study found no difference in the kinematics of the pelvis, hip or knee during the single-leg squat at 45° of knee flexion between individuals with and without PFOA [25]. However, the authors of the study evaluated the kinematics of the pelvis and lower limb only at this specific angle of knee flexion. As the movement pattern can change throughout the knee flexion range, the kinematics of the pelvis and lower limb should also be evaluated at other points of this range.

An important limitation of previous studies on segment/joint kinematics in individuals with PFOA is the failure to evaluate trunk movement. As stated above, previous studies found that individuals with PFP have excessive ipsilateral lean of the trunk during functional tasks involving weight bearing, such as the single-leg squat and the stepping task [17,18]. This excessive ipsilateral trunk lean may occur as compensation for contralateral pelvic drop due to weakness of the hip abductors [21]. In turn, excessive ipsilateral trunk lean results in an external abductor moment at the knee, increasing the patellofemoral load [17,18].

The strength of the hip abductors is lower in individuals with isolated PFOA [26–28]. It is known that the hip abductors are involved in the control of the movements of the pelvis, hip and knee on the frontal plane [17,18,29]. Thus, it is important to determine whether the kinematics of these segments/joints on the frontal plane are altered in this population at different points of the knee flexion range during the single-leg squat. A better understanding of this aspect could assist in the planning of rehabilitation programs for individuals with PFOA.

Therefore, the aim of the present study was to compare frontal plane trunk, pelvis, hip and knee motion at 30°, 45° and 60° of knee flexion during the descending and ascending of the single-leg squat, and isometric hip abductor strength between individuals with and without isolated PFOA. We hypothesized that individuals with isolated PFOA would exhibit greater ipsilateral trunk lean, contralateral pelvic drop, hip adduction and knee abduction and would have weaker isometric hip abductor strength.

## Materials and methods

### Study design

The present cross-sectional was conducted at the Rheumatology and Hand Rehabilitation Research Lab of the Physical Therapy Department of *Universidade Federal de São Carlos* (UFSCar), Brazil. This study followed the recommendations of the STROBE statement [30] and received approval from the UFSCar Human Research Ethics Committee (certificate number: 96324918.4.0000.5504). All participants signed a statement of informed consent. The data were collected between November 2018 and July 2019.

### Participants

All participants in this study resided in the city of São Carlos and were recruited through a call posted on the website of the institution and announcements on flyers as well as local radio stations, newspapers and magazines. All volunteers were submitted to a radiological exam of both knees and the severity of knee OA was graded using the Kellgren and Lawrence (KL) criteria [31]. The diagnosis of OA was based on the clinical and radiographic classification criteria of the American College of Rheumatology [32]. Three views of the knee were obtained for each subject: the weight-bearing anteroposterior, a skyline and a lateral view. The last two views were obtained in the supine position with the knee flexed to 45°. The patellofemoral joint was assessed using a skyline and a lateral views. PFOA was defined by a KL score ≥ 2 on the skyline view and/or the presence of a definite superior and/or inferior osteophyte on the patella surface of the lateral view [33]. The evaluation of the radiographs was performed by the same evaluator with 16 years of experience. Kappa coefficients were used to determine the test-retest reliability of KL scores. Kappa was 0.92 (95% confidence interval = 0.78–1.07).

Men and women between 40 and 65 years of age composed the sample and were divided into two groups: PFOA group and control group of healthy individuals. The inclusion criteria for the PFOA group were reported anterior patellar or retro-patellar pain of at least 4 on the 11-point numerical pain scale ranging from 0 (absence of pain) to 10 (worst pain possible) aggravated by two or more activities involving load on the patellofemoral joint, such as climbing stairs, standing up from a sitting position or squatting [24], morning stiffness lasting less than 30 minutes, joint crepitus [32], evidence of the formation of osteophytes in the patellofemoral joint in lateral and skyline axial views (Grade 2 or 3 of KL classification) [34], and ability to perform a single-leg squat to at least 60° of knee flexion. Individuals with unilateral or bilateral symptoms were included in the study. For inclusion in the control group, the individuals could not have any radiographic abnormalities of the knees, not have had lower limb pain in the previous six months and have the ability to perform a single-leg squat to at least 60° of knee flexion.

The exclusion criteria were those described by Pohl et al. [28] and applied to both groups: previous history of patella fracture or recurrent subluxation of the patella, bone abnormalities (fracture, osteochondritis dissecans, bipartite patella, etc.), known OA in other weight bearing joints (including spine), hip, knee or ankle osteotomy or arthroplasty, arthroscopic surgery or infiltration in the knee in the previous three months, having undergone physiotherapy in the previous six months, use of a cane or other gait-assistance device and any physical or medical problem considered a contraindication for the evaluations. Individuals with concomitant tibiofemoral OA (KL grade ≥ 2 on anteroposterior radiograph) were also excluded [35]. The two groups were matched for sex and physical activity level (physically active and sedentary).

### Procedures

The dominant leg was evaluated in the control group, which was determined by the answer to the following question: “What leg do you use to kick a ball a far as possible?” [36]. The affected limb was evaluated in the PFOA group. In cases of bilateral PFOA, the more symptomatic limb was evaluated (higher pain level determined using the numerical rating scale) [37]. The participants were instructed not to perform any type of physical activity beyond habitual activity in the 48 hours prior to the test.

Physical activity level was classified following the guidelines of the World Health Organization [38]. Participants who practiced 150 to 300 minutes of aerobic activity of moderate intensity, 75 to 150 minutes of vigorous aerobic activity or an equivalent combination of moderate and vigorous aerobic activity during the week for substantial health benefits were considered physically active. Those who practiced an intensity lower than that recommended by the WHO, were classified as sedentary.

The Western Ontario and McMaster Universities Osteoarthritis Index (WOMAC) in its version translated and validated for Brazilian Portuguese language was used to characterize the sample [39,40]. The WOMAC is a self-report questionnaire for patients with knee and/or hip OA and is divided into three domains: pain, stiffness and functional limitation. The domains are scored on a five-point scale (none = 0, mild = 1, moderate = 2, intense = 3 and very intense = 4). The maximum score for each domain of the questionnaire was considered, with higher scores denoting worse pain, stiffness and physical function.

#### Kinematic evaluation of single-leg squat

The Vicon motion capture and analysis system (Vicon Motion Systems Ltd, Oxford, UK) and the Nexus System 2.1.1 (Vicon Motion Systems Ltd, Oxford, UK) and 3D Motion Monitor (Innovative Sports Training Inc., Chicago, USA) were used for the acquisition and analysis of the kinematic data. Six Bonita 10 cameras (Vicon Motion Systems Ltd, Oxford, UK) were used to capture the trajectory of the markers at a sampling frequency of 90 Hz.

A single researcher positioned 28 reflective markers (14 mm in diameter) on the following anatomic landmarks of each volunteer: jugular notch, both acromial processes, spinous process of seventh cervical and tenth thoracic vertebrae, iliac crest (bilaterally), anterior superior iliac spine and posterior superior iliac spine (bilaterally), first sacral vertebra, greater trochanter (bilaterally), medial and lateral femoral condyles (bilaterally), medial and lateral malleoli (bilaterally), immediately over second metatarsal head on the shoe (bilaterally), immediately over calcaneus on the shoe (bilaterally) and lateral side of the foot on the shoe (on both feet but at different distances–immediately over the fifth metatarsal head on the right foot and base of the fifth metatarsal on the left foot). Moreover, four clusters (each comprising four non-collinear markers affixed to a rigid base) were attached to the participants using Velcro straps on the lateral face of the thigh and leg bilaterally. The participants were evaluated wearing shorts, a top (women) and athletic shoes (Asics model *GEL Equation 5*), which were provided by the researcher. A static reading of each participant in the neutral position was used to align the participants with the system of coordinates of the laboratory, serving as the point of reference for the subsequent kinematic analysis.

For the kinematic evaluation, the participants were instructed to squat to greater than 60° of knee flexion in a period of two seconds and return to the initial position in another period of two seconds (measured using a metronome) [18]. To achieve the established knee flexion angle, an adjustable support was placed beside the participants at a height that represented the distance from the floor to the greater trochanter of the required femoral mark [41]. The repetition was considered valid when the participant performed the single-leg squat with knee flexion of at least 60° within a period of four seconds without losing balance [18]. If the repetition was not considered valid, an additional repetition was performed. Five valid repetitions were collected for the analysis, with a one-minute rest period respected between repetitions. Prior to the test, each participant performed the squat for familiarization.

#### Evaluation of isometric hip abductor torque

Isometric hip abduction torque was measured using a handheld dynamometer (Lafayette Manual Muscle Test System, Lafayette Instruments, Lafayette, IN, USA). The participants were positioned as described by Nakagawa et al. [18] The participant remained in lateral decubitus on the examining table with the tested lower limb on top. A cushion was positioned between the legs so that the hip of the tested limb remained at approximately 10° of abduction [42]. A non-elastic strap was positioned immediately above the iliac crest and attached firmly around the examining table to stabilize the trunk. The dynamometer was positioned 5 cm proximal to the lateral joint line of the knee and was attached with a second non-elastic strap positioned around the leg and the examining table [18]. A command was given during the evaluation for the participant to perform maximum strength to raise the leg [19]. Prior to the trial, three submaximal isometric contractions and one maximum isometric contraction were performed for familiarization [18]. The maximum voluntary isometric contractions (peak value recorded in kilograms) were performed for five seconds each, with a two-minute rest between trials [43].

Prior to the study, to establish test-retest reliability of isometric hip abduction torque measurement, eight participants were tested on two occasions separated by three to five days. The intraclass correlation coefficient (ICC_3,1_) and standard error of measurement were 0.97 and 0.95 Nm/kg for hip abduction torque.

### Analysis of kinematic data

Kinematic data were processed using the 3D Motion Monitor (Innovative Sports Training, Chicago, IL, USA) software. The kinematic data were filtered using a 4th-order, zero-lag, low-pass Butterworth filter at 12 Hz.[44] The Euler angles were calculated using the joint coordinate system definitions recommended by the International Society of Biomechanics relative to the static standing trial [45,46]. Hip and knee kinematics were calculated as the motion of the distal segment relative to the proximal reference. Pelvis and trunk angles were calculated as the motion of the segment relative to the global coordinate system. The center of the knee was defined as the midpoint between the medial and lateral epicondyles. The center of the hip was determined using the method described by Bell, Pedersen and Brand [47].

The analysis of the kinematic variables was performed using a custom program in Matlab (Mathworks, Natick, MA, USA). The variables of interest were ipsilateral (+)/contralateral (−) trunk lean, contralateral pelvic elevation (+)/drop (−) and abduction (+)/adduction (−) of the hip and knee at 30°, 45° and 60° of knee flexion in both the descending and ascending phases of the single-leg squat.

For the isometric hip abductor torque, the results of the all trials [kg] were converted into Newtons (strength [N] = strength [kg] x 9.81) to determine a unit of force and Newtons were then converted into torque (torque [Nm] = force [N] x action length [m]) [48]. The length between the greater trochanter and the lateral epicondyle of the femur was used as the action length. All torque (Nm) data were normalized by body mass (normalized torque [Nm/kg] = torque (Nm) ÷ body mass [kg]). For statistical purposes, the average of three trials with less than 10% variability was considered. When a difference greater than 10% occurred between trials, a fourth trial was performed [49].

### Statistical analysis

The sample size was calculated with the aid of the G*Power software (version 3.1.9.2; Kiel University, Germany) based on the hip adduction angle at 60° of knee flexion in single-leg squat of the first four participants in each group. Considering a significance level of α = 0.05 and β = 0.95 to detect a difference in hip adduction angle of 15.2° with a standard deviation (SD) of 7.1, six participants would be needed for each group.

The data were analyzed with the aid of IBM SPSS Statistics for Windows, Version 25.0. (IBM Corp., Armonk, NY, USA). Normality and homoscedasticity were determined using the Shapiro-Wilk and Levene tests, respectively. Data with non-normal distribution were log-transformed (angles of trunk lean at 60° of knee flexion and hip abduction/adduction at 30° of knee flexion in descending phase of single-leg squat; trunk lean at 60° and 45° of knee flexion and hip abduction/adduction at 45° of knee flexion in ascending phase of single-leg squat). For the kinematic variables, mixed two-way ANOVA (group*knee flexion angle) was performed considering knee flexion angle as repeated measures. The Bonferroni test was applied when significant differences were detected. The Student’s t-test for independent variables was used for the comparison of demographic and anthropometric variables and for isometric hip abductor torque. The Mann-Whitney U-test was used for the comparison between groups regarding the WOMAC domain scores to characterize the sample. The effect size (Hedges’ g) was calculated for each comparison using Cohen’s classification [50] for the interpretation of the standard mean difference, with values of 0.8, 0.5 and 0.2 corresponding to large, medium and small effect sizes, respectively. For all analyses, the level of significance was set at 5% (p ≤ 0.05).

## Results

From a list of 108 individuals, 88 were excluded based on the exclusion criteria or failed to return for the subsequent assessments. Of these, 44 individuals had mixed OA (in patellofemoral and tibiofemoral [grade ≥ 2] compartments), 12 had isolated PFOA grade 1, and three had isolated tibiofemoral OA. Twenty participants matched the eligibility criteria. The demographic and anthropometric characteristics of the groups are displayed in Table 1. Nine of the ten participants in the PFOA group had isolated PFOA. Only one participant in the group had doubtful OA (KL grade 1) in the tibiofemoral compartment. No significant differences between groups were found regarding age or body mass index (p > 0.05). The two groups were similar in terms of physical activity level. In comparison to the control group, the PFOA group had higher scores on all WOMAC domains (p ≤ 0.002) (Table 1).

**Table 1:** Demographic and clinical characteristics of patellofemoral osteoarthritis group and control group.

	Mean ± SD		Median (IQR)					
Characteristics	Patellofemoral Osteoarthritis (n = 10)	Control (n = 10)	Patellofemoral Osteoarthritis (n = 10)	Control (n = 10)	Mean difference (95% CI)	p-value	Mann-Whitney U	Effect size
Age (years)	51.8 ± 6.9	47.8 ± 5.5	-	-	4 (-1.9 to 9.9)	0.20	-	0.61
BMI (kg/m^2^)	27.6 ± 2.2	25.5 ± 2.5	-	-	2.1 (-0.1 to 4.3)	0.06	-	0.85
Female (n; %)	5 (50)	5 (50)	-	-	-	-	-	-
Male (n; %)	5 (50)	5 (50)	-	-	-	-	-	-
Physical activity level^a^ (n; %)								-
Active	7 (70)	7 (70)	-	-	-	-	-	-
Sedentary	3 (30)	3 (30)	-	-	-	-	-	-
Kellgren & Lawrence classification	Grade II = 7 Grade III = 3	Grade 0 = 10	-	-	-	-	-	-
WOMAC scores^b^								
Pain^c^	2.2 ± 2.7	0.2 ± 0.4	1 (4.25)	0 (0.25)	2.0 (0.19 to 3.81)	0.04*^,^^f^	26,000	0.99
Stiffness^d^	1.5 ± 1.7	0.2 ± 0.4	1 (3.25)	0 (0.25)	1.3 (0.14 to 2.46)	0.04*^,^^f^	26,000	0.12
Physical function^e^	8.2 ± 7.8	0.9 ± 2.5	8 (10.5)	0 (0.25)	7.3 (1.86 to 12.72)	0.002*^,^^f^	10,500	1.21

^a^Physical activity level according to World Health Organization[38]

^b^Median and interquartile range for WOMAC domain scores

^c^Range of possible scores: 0 to 20

^d^Range of possible scores: 0 to 8

^e^Range of possible scores: 0 to 68

^f^p-value for Mann-Whitney U test

*Significant difference: p ≤ 0.05.

Abbreviations: BMI: Body mass index; IQR: Interquartile Range; SD: Standard deviation; WOMAC, Western Ontario and McMaster Universities Osteoarthritis Index.

The results of the kinematic analysis are presented in Table 2. The individuals with isolated PFOA had a significantly larger hip adduction angle during the single-leg squat at 45° (mean difference [95% CI] = 6.44° [0.39–12.48°]) and 60° (mean difference [95% CI] = 8.44° [2.15–14.73°]) of knee flexion in the descending phase as well as at 60° (mean difference [95% CI] = 7.58° [2.1–13.06°]) and 45° (mean difference [95% CI] = 5.33° [0.24–10.42°]) of knee flexion in the ascending phase. No significant differences between groups were found regarding trunk lean, pelvic elevation or knee abduction at 30°, 45° and 60° of knee flexion in the descending or ascending phases (p > 0.05). The PFOA group presented lower isometric hip abductor torque compared to the control group (mean difference [95% CI] = -0.34 Nm/kg [-0.67 to -0.01 Nm/kg]), exhibiting an average of 21.9% less isometric hip abductor torque.

**Table 2:** Between-group comparisons of frontal plane joint angles during single-leg squat (in degrees) and normalized isometric hip abductor torque (Nm/kg).

Variables	Group		Mean difference (95% CI)	p-value	Effect size
	Patellofemoral Osteoarthritis (n = 10)	Control (n = 10)			
*Kinematics (Descending phase)*					
Ipsilateral trunk lean (+)/Contralateral (−)					
Knee flexion at 30°	3.61±3.23	2.95±2.34	0.66 (-1.99 to 3.31)	0.61	0.22
Knee flexion at 45°	4.27±3.71	3.57±2.47	0.7 (-2.26 to 3.66)	0.63	0.21
Knee flexion at 60°	5.69±5.04	4.61±3.17	1.08 (-2.88 to 5.04)	0.94	0.25
Pelvic elevation (+)†/drop (−)					
Knee flexion at 30°	2.59±2.92	2.95±2.14	-0.36 (-2.78 to 2.04)	0.75	0.13
Knee flexion at 45°	1.99±2.8	2.79±2.73	-0.8 (-3.4 to 1.8)	0.53	0.30
Knee flexion at 60°	0.75±4.18	2.35±3.16	-1.6 (-5.01 to 1.89)	0.35	0.41
Hip abduction (+)/adduction (−)					
Knee flexion at 30°	−7.99±7.53	−3.89±3.99	4.10 (-1.56 to 9.76)	0.23	0.65
Knee flexion at 45°	−10.04±7.55	−3.60±5.06	6.44 (0.39–12.48)	0.04*	0.96
Knee flexion at 60°	−14.47±7.29	−6.03±6.05	8.44 (2.15–14.73)	0.01*	1.21
Knee abduction 0028+)/adduction (−)					
Knee flexion at 30°	9.02±5.07	10.60±5.81	-1.58 (-6.7 to 3.55)	0.53	0.28
Knee flexion at 45°	16.0±6.35	17.46±9.02	-1.46 (-8.79 to 5.87)	0.58	0.18
Knee flexion at 60°	23.26±8.82	21.46±9.73	1.8 (-6.93 to 10.52)	0.67	0.19
*Kinematics (Ascending phase)*					
Ipsilateral trunk lean (+)/Contralateral (−)					
Knee flexion at 60°	4.89±6.96	5.28±3.28	-0.39 (-5.50 to 4.72)	0.6	0.07
Knee flexion at 45°	4.36±5.94	4.18±2.93	0.18 (-4.22 to 4.58)	0.48	0.04
Knee flexion at 30°	3.92±4.97	3.49±2.39	0.43 (-3.23 to 4.09)	0.81	0.11
Pelvic elevation (+)†/drop (−)					
Knee flexion at 60°	1.1±4.7	2.16±4.63	-1.06 (-5.44 to 3.32)	0.61	0.22
Knee flexion at 45°	1.5±3.51	2.45±3.92	-0.95(-4.45 to 2.55)	0.59	0.25
Knee flexion at 30°	2.43±2.33	2.88±3.01	-0.45 (-2.98 to 2.08)	0.71	0.16
Hip abduction (+)/adduction (−)					
Knee flexion at 60°	−16.86±6.77	−9.28±4.71	7.58 (2.1–13.06)	0.009*	1.25
Knee flexion at 45°	−12.15±6.44	−6.82±4.15	5.33 (0.24–10.42)	0.045*	0.94
Knee flexion at 30°	−8.54±5.45	−5.29±3.48	3.25 (-1.05 to 7.55)	0.13	0.68
Knee abduction (+)/adduction (−)					
Knee flexion at 60°	22.97±10.1	19.24±8.76	3.73 (-5.14 to 12.61)	0.39	0.40
Knee flexion at 45°	16.93±7.97	15.4±8.81	1.53 (-6.36 to 9.42)	0.69	0.17
Knee flexion at 30°	9.68±5.35	9.03±6.45	0.65 (-4.92 to 6.21)	0.81	0.11
*Torque*					
Isometric hip abductor	1.21 ± 0.30	1.55 ± 0.39	-0.34 (-0.67 to -0.01)	0.04*	0.94

*Significant difference: p ≤ 0.05.

†Larger value = less contralateral pelvic lean.

## Discussion

The aim of the present study was to compare the frontal plane kinematics of the trunk, pelvis, hip and knee at 30°, 45° and 60° of knee flexion during the descending and ascending phases of the single-leg squat and isometric hip abductor torque between individuals with and without isolated PFOA. The results of this study partially confirmed the hypotheses, demonstrating that individuals with symptomatic radiographic PFOA present larger hip adduction angles at 45° and 60° of knee flexion in both the descending and ascending phases of the single-leg squat and have less capacity to generate isometric hip abductor torque in comparison to individually matched controls. Nakagawa et al. [18] reported similar results for individuals with PFP during the descending and ascending phases of the stepping task compared to control subjects. This finding is important, as excessive hip adduction and knee abduction are the main components of dynamic knee valgus in the frontal plane [22] and an increase in dynamic knee valgus results in an increase in the quadriceps angle (Q angle), with a consequent increase in lateralizing forces that act on the patella, leading to greater stress on the lateral patellofemoral joint [21]. Larger hip adduction angles in individuals with PFOA have also been found in the late support phase of the gait cycle [24].

In contrast, Macri et al. [25] found no significant differences in hip adduction angles during the single-leg squat at 45° of knee flexion between individuals with and without PFOA. This divergence may be due to differences in the evaluation systems used. A three-dimensional motion analysis system was used in the present investigation, whereas Macri et al. [25] used a two-dimensional system to estimate the alignment of the pelvis, hip and knee on the frontal plane. The eligibility criteria also differed between the two studies. Macri et al. [25] included individuals with Grade ≥ 1 PFOA according to the KL classification (individuals with radiographic proof of at least doubtful narrowing of the joint space with the possible formation of osteophytes), whereas only individuals with Grade 2 or 3 PFOA were included in the present study (radiographic proof of definite osteophyte formation and possible or definite narrowing of the joint space with some sclerosis and possible deformity of the bone extremities). Moreover, Macri et al. [25] did not mention the relative and absolute frequency of the degrees of PFOA among the individuals included in the study. Finally, the individuals with PFOA in the study by Macri et al. [25] did not present a reduction in isometric hip abduction strength compared to the control subjects. As the hip abductor muscles act eccentrically to control adduction of this joint during the single-leg squat, the strength deficit found in the present study may be associated with the greater hip adduction in the PFOA group. Such factors may explain the divergence in the findings between the two studies.

Contrary to our hypothesis, no significant differences between groups were found regarding the kinematics of the trunk and pelvis. We had hypothesized that individuals with isolated PFOA would present greater contralateral pelvic drop and greater ipsilateral trunk lean, as found in a previous study involving individuals with PFP during the single-leg squat and the stepping task [17,18]. Excessive ipsilateral trunk lean may be compensation for the weakness of the hip abductor muscles [21]. However, although the PFOA group in the present study exhibited a 21.9% deficit in isometric hip abductors torque, this deficit did not result in changes in the kinematics of the pelvis or trunk. It is possible that greater deficits in hip abduction strength are necessary before the occurrence of changes in trunk and pelvis kinematics during the single-leg squat. Pohl et al. [28] also found no difference between groups with and without PFOA regarding peak contralateral pelvic drop during gait on a treadmill. In contrast, Crossley et al. [24] found that individuals with PFOA exhibited greater contralateral pelvic drop during the late support phase of the gait cycle. As the authors did not evaluate hip abductor strength, it is not possible to determine whether the individuals with PFOA in the study by Crossley et al. [24] exhibited greater strength deficit compared to those in the present study, which could result in excessive contralateral pelvic drop.

Also contrary to our hypotheses, no difference between groups was found regarding the movement of the knee in the frontal plane. In contrast, Hoglund et al. [23] found greater knee abduction in individuals with PFOA during the tasks of sitting and standing. The movement of the trunk on the frontal plane can alter the load and position of the knee. Excessive ipsilateral trunk lean displaces the vector of the ground reaction force laterally to the knee, producing an external knee abduction moment [21], which can contribute to an increase in abduction in this joint [51,52]. Therefore, the lack of a difference in the trunk movement on the frontal plane may explain the lack of a difference between groups regarding the knee movement found in the present study.

To the best of our knowledge, this is the first study to evaluate frontal plane trunk, pelvis, hip and knee motion at different angles of knee flexion during the single-leg squat in individuals with and without PFOA. It is important to recognize kinematic changes in the trunk, pelvis, hip and knee at different angles of knee flexion during the descending and ascending phases of a given functional task, such as the single-leg squat, to design more specific and effective treatment protocols for individuals with PFOA. For instance, the present study showed that individuals with isolated PFOA have lower isometric hip abductor torque and an increase in adduction in this joint, suggesting that it would be potentially beneficial to include strengthening exercises targeting the hip abductors in the treatment of such individuals. A recent viability study found that a six-week program of core and hip muscle strengthening for individuals with PFOA can reduce pain in the short term and this improvement can be maintained for at least six months [53]. However, large randomized controlled trials are needed for a better understanding of the effects of hip muscle strengthening programs on pain and function in the long term for this population.

This study has limitations that should be considered. The sample size may have led to the lack of differences between groups regarding the other kinematic variables of the trunk, pelvis and knee (type II error). Thus, future studies with a larger sample size may find differences between groups for the other variables. The cross-sectional design limits the ability to establish cause-and-effect relationships. Therefore, prospective studies are needed to enable drawing definitive conclusions regarding the role of the kinematics of the trunk, pelvis, hip and knee as well as hip muscle strength in individuals with PFOA. Previous studies have shown that individuals with PFP have altered hip and knee kinematics in the transverse plane as well as strength deficits regarding hip extension and external rotation. The present study only evaluated the hip and knee kinematics on the frontal plane and hip abduction strength. Thus, future studies should evaluate the kinematics of the hip and knee on the transverse plane at different angles of knee flexion during the single-leg squat as well as hip extension and external rotation strength. Moreover, the kinematics of the trunk, pelvis, hip and knee in individuals with isolated PFOA should be evaluated during the execution of other functional tasks.

## Conclusions

The individuals with isolated PFOA presented increased hip adduction at 45° and 60° of knee flexion in the both descending and ascending phases of the single-leg squat in comparison to healthy controls. The individuals with isolated PFOA also exhibited less capacity to generate hip isometric abductor torque. The results of this study suggest that hip abductor strengthening and motor control training with a focus on frontal plane hip alignment may be appropriate in the management of PFOA.
